# Cardiogenic shock elicits acute inflammation, delayed eosinophilia, and depletion of immune cells in most severe cases

**DOI:** 10.1038/s41598-020-64702-0

**Published:** 2020-05-06

**Authors:** Justine Cuinet, Andrea Garbagnati, Marco Rusca, Patrick Yerly, Antoine G. Schneider, Matthias Kirsch, Lucas Liaudet

**Affiliations:** 1 Service of Adult Intensive Care Medicine, University Hospital Medical Center and Faculty of Biology and Medicine, Lausanne, Switzerland; 2 Service of Cardiology, University Hospital Medical Center and Faculty of Biology and Medicine, Lausanne, Switzerland; 3 Service of Cardiac Surgery, University Hospital Medical Center and Faculty of Biology and Medicine, Lausanne, Switzerland

**Keywords:** Immunology, Cardiology

## Abstract

Patients with cardiogenic shock (CS) display systemic inflammation and a high rate of infections, suggesting important immune disturbances. To explore the immune response to CS, we prospectively measured, in 24 consecutive CS patients, differential white blood cell (WBC) counts and the cytokines IL-1β, IL-5, IL-6, IL-10, TNFα, IFNγ, MCP-1 and eotaxin (CCL11), at Day 1 (T1), day 3 (T2) and day 6-8 (T3). Secondary infections and their influence on cytokines and WBCs were determined. CS induced early (T1) neutrophilia and elevated levels of IL-6, IL-10 and MCP-1, correlating with shock severity. The eosinophil chemoattractant eotaxin was elevated at T1 and decreased thereafter, and a progressive rise of blood eosinophils was noted over time. Patients with the most severe shock had reduced lymphocytes and monocytes at T2 and T3. Sixty-two percent of patients developed an infection, which did not alter the profile of immune response, except from higher IL-6 levels at T2. Therefore, CS elicits an acute pro-inflammatory response, followed by a delayed increase in blood eosinophils, consistent with the development of a tissue repair response, as well as depletion of immune cells in the most severely affected patients, which might predispose to secondary infections.

## Introduction

Cardiogenic shock (CS) defines a state of systemic hypoperfusion leading to end-organ dysfunction related to cardiac pump failure primarily caused by acute myocardial infarction, and with mortality rates in the range of 27–51% according to recent reviews^1^. The high mortality of CS likely reflects its complex, and incompletely understood pathophysiological mechanisms. While the acute compromise of central hemodynamics promotes systemic impairment of cellular oxygenation, additional drivers of tissue malperfusion and cytotoxicity may also play critical roles. Many patients with CS display clinical signs of systemic inflammation^2^ and elevated plasma levels of prototypical inflammatory mediators, such as C-reactive protein (CRP), Interleukin-6 (IL-6) and tumor necrosis alpha (TNFα)^3,4^. Furthermore, a significant proportion of CS patients develop secondary infectious complications^5,6^, which could reflect a state of impaired antimicrobial defenses. These characteristics suggest the hypothesis that a state of disordered immunity stands at the foreground of the pathophysiology of CS and could represent a potential therapeutic target in this condition. Only few studies have investigated immune alterations in the setting of CS, and most have been restricted to the measurements of inflammatory cytokines, primarily IL-6 and TNFα, at the acute phase of CS^4,7,8^. Moreover, although an initial increase in white blood cell (WBC) count represents an important risk factor for poor outcome in patients with myocardial infarction^9^, the behavior of different WBC populations following CS has not been evaluated. We therefore sought to explore in more detail the immune response to CS, by performing sequential measurements during the first week of hospitalization of a panel of cytokines, of differential counts of WBCs, and of the infectious complications in a series of 24 patients hospitalized for CS.

## Results

Patients’ characteristics are shown in Table 1. Patients were predominantly males (79%), with a median (IQR) age of 63 (19) years. The admission median (IQR) left ventricle ejection fraction (LVEF) was 20% (15%) and the most frequent causes of CS were acute coronary syndrome (12 pts, 50%) and decompensation of chronic cardiomyopathy (6 pts, 25%). The in-hospital mortality was 21% (5 pts/24). All patients received catecholamines, including norepinephrine (23/24 pts, median dose of 0.32 mg/h, IQR 0.52 mg/h) and dobutamine (19/24 pts, median dose of 8.1 mg/h, IQR 11.7 mg/h) (first 48 h). An intra-aortic balloon pump was inserted in 9 pts (38%) and a veno-arterial extra-corporeal membrane oxygenator (VA-ECMO) in 4 pts (17%).

**Table 1:** Study population characteristics.

Patients, n	24
Age (yr), median (IQR)	63 (19)
Male, n (%)	19 (79)
Hypertension, n (%)	10 (40)
Type 2 diabetes, n (%)	6 (25)
Previous MI, n (%)	9 (36)
COPD, n (%)	10 (40)
Chronic heart failure, n (%)	6 (25)
LV EF on admission (%), median (IQR)	20 (15)
Admission arterial blood lactate (mmol/L), median (IQR)	3.4 (2.7)
APACHE II score, median (IQR)	21 (12)
SAPS II score, median (IQR)	51 (22)
SOFA score (first 24 h), median (IQR)	10 (4)
ICU LOS (d), median (IQR)	6.3 (6.9)
Hospital LOS (d), median (IQR)	21 (29)
In-Hospital mortality, n (%)	5 (21)
**Etiology of CS**	
Acute coronary syndrome, n (%)	12 (50)
Decompensated chronic cardiomyopathy, n (%)	6 (25)
Post-CPB, n (%)	2 (8)
Others *, n (%)	4 (16)
Urgent coronary angiography, n (%)	18 (75)
**Hemodynamic therapy, first 48 h**	
Norepinephrine, n (%) **	23 (96)
Dobutamine, n (%) ***	19 (79)
Epinephrine, n (%)	4 (17)
Vasopressine, n (%)	2 (8)
Milrinone, n (%)	1 (4)
Levosimendan, n (%)	8 (33)
IABP, n (%)	9 (38)
VA-ECMO, n (%)	4 (17)
Invasive mechanical ventilation, n (%)	20 (83)
Hemodynamic monitoring, n (%)	16 (67)
Pulmonary artery catheter, n (%)	15 (63)
Transpulmonary thermodilution (PiCCO®), n (%)	1 (4)
No invasive monitoring, n (%)	8 (33)

*Myocarditis (n = 1); Takotsubo cardiomyopathy (n = 1); Acute aortic valve insufficiency (n = 1); Ventricular tachycardia (n = 1).

**Norepinephrine, median (IQR) dose first 48 h: 0.32 (0.52) mg/h.

***Dobutamine, median (IQR) dose first 48 h: 8.1 (11.7) mg/h.

APACHE, Acute Physiology And Chronic Health Evaluation; COPD: Chronic Obstructive Pulmonary Disease; CPB: Cardiopulmonary Bypass; IABP: Intra-Aortic Balloon Pump; ICU, Intensive Care Unit; LOS, Length Of Stay; LVEF, Left Ventricle Ejection Fraction; SAPS: Simplified Acute Physiology Score; SOFA: Sequential Organ Failure Assessment; VA-ECMO: Veno-Arterial Extra-Corporeal Membrane Oxygenation.

Hemodynamic variables are shown in Table 2. Heart rate (HR) and diastolic blood pressure (BP) did not change significantly from admission to T3, while mean BP, systolic BP and cardiac index (CI) displayed a significant increase at T2 and T3 as compared to admission values. Laboratory chemical variables are shown in Table 3. Biomarkers of myocardial damage (troponin, NT-pro-BNP), liver injury (AST, ALT) and kidney damage (creatinine) were elevated at T1, and non-significantly decreased over time. The low arterial pH and high plasma lactate at T1 returned to normal values thereafter. Plasma procalcitonine levels did not vary significantly over time.

**Table 2:** Hemodynamic data.

Variable	Admission (n = 24)	T1 (n = 24)	T2 (n = 23)	T3 (n = 15)
Heart rate (bpm)	84 (23)	92 (24)	87 (26)	89 (16)
Systolic BP (mm Hg)	89 (15)	98 (14)	100 (14)*	112 (39)*
Diastolic BP (mm Hg)	54 (12)	58 (13)	57 (7)	58 (15)
Mean BP (mm Hg)	63 (8)	70 (12)	72 (9) *	70 (22) *
Cardiac index (ml/min/m^2^)	1.6 (0.7)	2.3 (1.1)	2.6 (1.0) *	2.8 (0.6) *

All data are medians (IQR). *P < 0.05 vs admission values (ANOVA and Tukey post hoc test)

For Cardiac index, n were: admission: 12; T1: 15; T2: 12; T3: 7

**Table 3:** Time course of laboratory values.

Variable	T1 (n = 24)	T2 (n = 23)	T3 (n = 15)
AST (UI/L)	267 (609)	211 (639)	72 (147)
ALT (UI/L)	96 (160)	73 (421)	96 (275)
Troponin T (μg/L)	1069 (4215)	455 (5362)	545 (4565)
NT-proBNP (ng/L)	9400 (28613)	6265 (19972)	7095 (11802)
Glucose (mmol/L)	9.4 (3.9)	6.9 (1.5)*	7.8 (1.9)
Creatinine (μmol/L)	121 (57)	97 (40)	74 (56)
pHa	7.33 (0.26)	7.42 (0.11)*	7.47 (0.08)*
Lactate (mmol/L)	3.4 (2.7)	1.2 (0.7)*	1.2 (0.4)*
CRP (mg/L)	44 (75)	128 (83)*	119 (69)*
Procalcitonine (μg/L)	0.48 (1.45)	0.63 (1.38)	0.54 (1.34)

For all measurements, n = 24 (T1), n = 23 (T2), and n = 15 (T3). Data are given as medians (IQR).

*p < 0.05 vs T1 (One way ANOVA and post hoc Tukey test).

**Figure 1:**
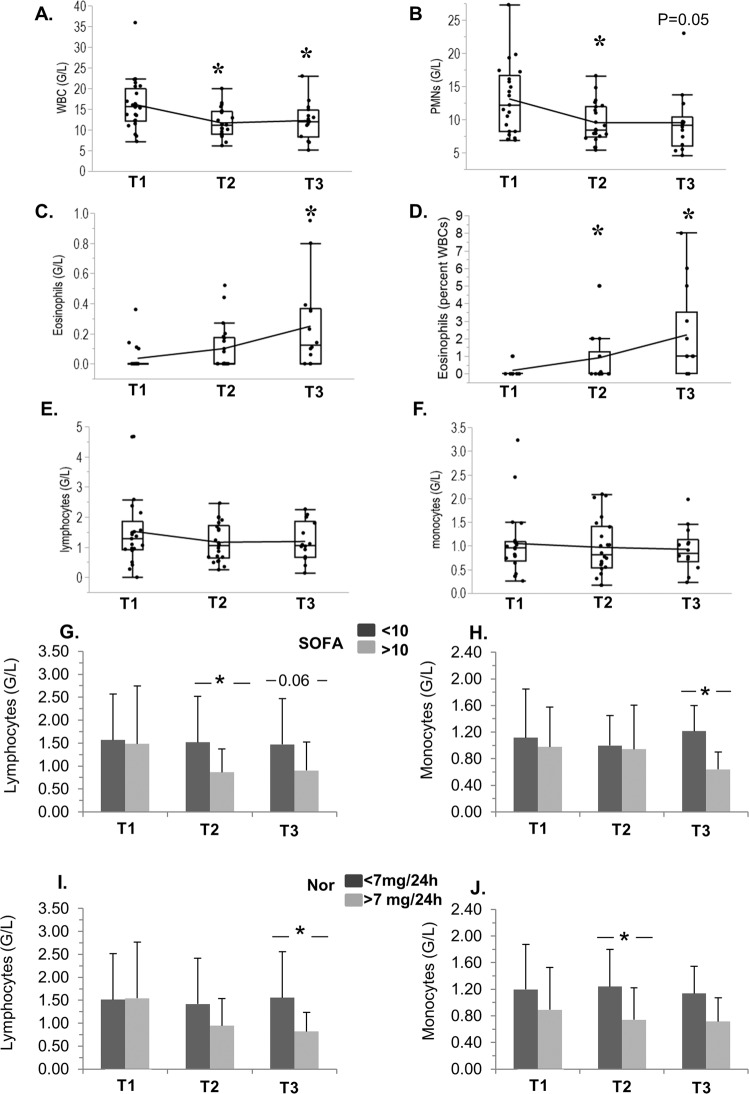
Time-course of white blood cells, and depletion of immune cells according to SOFA score and norepinephrine treatment, in cardiogenic shock. Evolution at T1 (day 1), T2 (Day 3), and T3 (Day 6-8) of WBCs (**A**), PMNs (**B**), Eosinophils (**C**: absolute numbers; **D** percent of total WBCs), Lymphocytes (**E**) and Monocytes (**F**). (**G**–**J)** Absolute numbers of Lymphocytes (left panel) and Monoyctes (right panel) at T1, T2 and T3 in patients separated according to their values of SOFA (**G**,**H**) and Norepinephrine dose in the first 24 h (**I**,**J**), above or below the median values of the whole cohort. The number of observations for different populations of WBCs was n = 21 (T1); n = 22 (T2) and n = 14 (T3). (**A**–**F**) Box plots show medians and IQRs. The means are joined by continuous lines. *p < 0.05 vs T1 (ANOVA and Tukey test). G-J: Means ± SDs. *p < 0.05 (Wilcoxon’s rank-sum test).

As shown in Fig. 1, the number of WBCs (Fig. 1A) and polymorphonuclear (PMNs) cells (Fig. 1B) decreased over time. In contrast, circulating eosinophils displayed a significant increase at T3 in comparison to T1 (Fig. 1C, absolute number, Fig. 1D percent of total WBCs). Lymphocytes and monocytes were comparable at all time-points in the whole cohort (Fig. 1E,F), but significant variations were noted when they were compared according to the severity score SOFA (Sequential Organ Failure Assessment) and norepinephrine administered (Fig. 1 G-J), although these effects were noted at different time-points: patients with higher SOFA had reduced lymphocytes at T2 and T3 (Fig. 1G), and less monocytes at T3 (Fig. 1H), whereas those with higher doses of norepinephrine had less lymphocytes at T3 (Fig. 1I) and monocytes at T2 (Fig. 1J).

**Figure 2:**
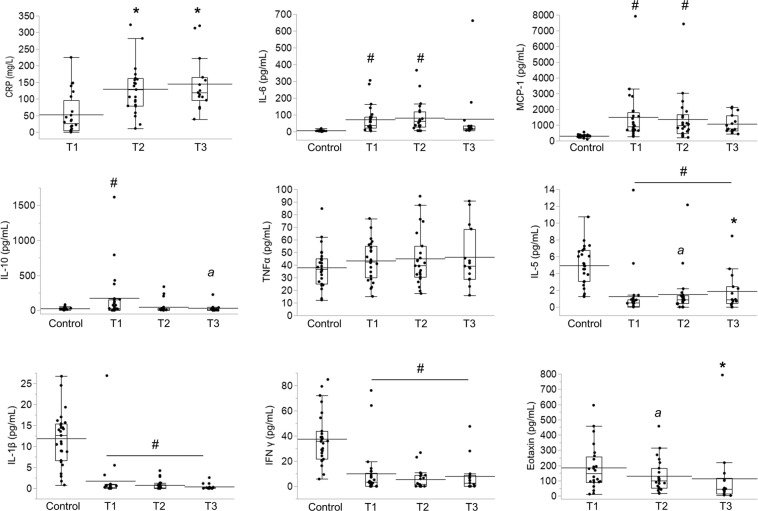
Time-course of plasma CRP and cytokines in cardiogenic shock. Data were obtained on day 1 (T1, n = 24), day 3 (T2, n = 23) and day 6-8 (T3, n = 15). Control values were obtained from hemodynamically stable patients scheduled for cardiac surgery (n = 30), except from eotaxin, which was measured only in CS patients. Box plots show medians and IQRs. The mean values are shown as straight bars. *p < 0.05 vs T1, *a*: p = 0.06 vs T1 (Wilcoxon’s rank-sum test). ^#^p < 0.05 vs Controls (Dunnett’s test).

The results of plasma C-reactive protein (CRP) and cytokines are shown in Fig. 2. CRP was elevated at T1, and displayed further significant increases at T2 and T3. Regarding cytokines, IL-6 and MCP-1 were significantly higher than in controls at T1 and T2, and did not display any significant variations over time. IL-10 was above control values at T1 only, and we noticed a slight decline of IL-10 at T3 (p = 0.06 vs T1). TNFα was not different from controls and did not vary over time. IL-5 was below control values, but displayed a progressive increase over time (at T2, p = 0.06 vs T1; at T3, p < 0.05 vs T1), while IFNγ and IL-1β were below control values and did not vary over time. It must be pointed out that control values for IL-5, IFNγ and IL-1β were relatively high, possibly reflecting the fact that these cytokines are known to be significantly higher when measured in EDTA plasma (as in the control group) than in heparinized plasma (as in our CS patients)^10^. The plasma levels of eotaxin (measured only in the group of CS patients) was highest at T1, and then progressively decreased over time (at T2, p = 0.06 vs T1; at T3, p < 0.05 vs T1). The results of correlation studies are shown in Table 4. At T1, mean BP negatively correlated with MCP-1, IL-6 and IL-10. NT-pro-BNP positively correlated with IL-6 at T1, and with MCP-1 at T2. The dose of norepinephrine (first 48 h) positively correlated with MCP-1, IL-6 and IL-10 at T1, and with MCP-1 at T2.

**Table 4:** Correlations between hemodynamic variables and cytokines.

Variable	Correlation	r^2^	p	direction of correlation
Mean BP T1	MCP-1 (T1)	0.294	0.008	negative
	IL-6 (T1)	0.293	0.006	negative
	IL-10 (T1)	0.4	0.0009	negative
NT-pro-BNP T1	IL-6 (T1)	0.346	0.004	positive
NT-pro-BNP T2	MCP-1 (T2)	0.479	0.0005	positive
Norepinephrine*	MCP-1 (T1)	0.173	0.04	positive
	MCP-1 (T2)	0.403	0.002	positive
	IL-6 (T1)	0.20	0.03	positive
	IL- 10 (T1)	0.24	0.01	positive
Dobutamine*	No correlations			

*Norepinephrine and Dobutamine: average hourly dose, first 48 h.

A significant proportion of patients (15/24, 62%) developed an infectious complication (Table 5) requiring antibiotic therapy, mostly from a pulmonary primary focus (11 pts). Most infections were microbiologically documented, except from 4 episodes, with Gram negative bacilli as the most frequent micro-organisms (13 specimens). Antibiotic therapy was started an average of 3.4 days after admission and for a mean duration of 8.5 ± 4.3 days. In addition to these 15 patients with de novo infection, 2 patients were already treated with antibiotics at inclusion, one for a suspected endocarditis, and one for a chronic osteomyelitis, and were not included in the calculation of the duration of antibiotic therapy. To determine whether infection could have influenced the time course of immune response, we compared WBCs and cytokines according to the presence or absence of infection. As indicated in Supplemental Table S1, the only statistically significant differences were different monocyte counts (higher at T1, p = 0.05, and lower at T2, p < 0.05), and higher IL-6 values at T2 (p = 0.05) in patients with an infection. We did not compare values at T3, since only one patient without infection was still in the ICU at T3.

**Table 5:** Infectious complications and antibiotic therapy.

Patients with a novel infection *, n (%)	15 (62%)
Primary source of infection	Lung (n = 11); Urinary tract (n = 1); Mixed lung + urinary tract (2); Mixed lung + primary bacteremia (1)
Type of microorganisms	*H. influenzae* (2), *S. pneumoniae* (2), *A. baumanii* (1), *C.koseri (1), S. aureus (1), E. cloacae (2), E. coli (3), K. pneumoniae (1), S. mitis (1); Unspecified enterobacterium (1); C.albicans (1); No documentation (4)*
Antibiotic therapy (days), mean ± SD	8.5 ± 4.3
First antibiotics (day after admission)	3.4 ± 1.9

In addition to the 15 patients with a novel infectious complication during their stay, 2 additional patients were receiving antibiotics at inclusion into the study (1 pt with a chronic osteomyelitis, 1 pt with a suspected endocarditis). These 2 patients were not included in the calculation of the duration of antibiotic therapy.

## Discussion

In the past 20 years, several investigators reported on an increase in the plasma levels of CRP^11^ and inflammatory cytokines, primarily IL-6 and TNFα in CS patients^4,7,8^, the intensity of this response being correlated with the severity of CS^4^. Moreover, many patients surviving the acute low cardiac output syndrome associated with CS display a profile of vasodilatory shock, with low systemic vascular resistance^2^ and normal or increased cardiac output^12^, consistent with the development of a systemic inflammatory response syndrome. Although the precise mechanisms responsible for triggering such inflammation in CS remain undefined, they may include innate immune activation in response to the release of damage associated molecular patterns (DAMPs or alarmins) by injured cells in ischemic tissues^13–15^, or the translocation of pathogen-associated molecular patterns (PAMPs), such as endotoxin, from the ischemic intestine^16^.

We confirm here that CS is associated with such acute inflammation, evidenced by high plasma CRP, increased WBC counts with neutrophilia, and elevated levels of IL-6, IL-10 and MCP-1. The increased concentrations of these cytokines correlated with the severity of circulatory failure, as indicated by their negative correlation with blood pressure, and positive correlation with norepinephrine treatment and NT-proBNP during the first 2 days, which is in full agreement with the above-cited reports showing a direct link between the degree of acute inflammation and the severity of hemodynamic deterioration in CS. In contrast, IL-1β and IFNy were not elevated, and even displayed lower values than in our control population, suggesting that these two cytokines do not play a major role in the acute phase of CS. At variance with IL-6 and MCP-1, IL-10 has purely anti-inflammatory effects, and could thus represent an early counter regulatory response, which is typical of the acute immune response in various forms of circulatory shock^17^. IL-10 is induced in monocytes by TNFα as an essential negative feedback mechanism to dampen inflammation^18^, and its release may also be a catecholamine-mediated process^19^. This would be consistent with the correlation found between norepinephrine administration and the initial concentration of IL-10. Thus, the early increase of IL-10 could either represent an attempt to limit early inflammation, or reflect instead the intensity of catecholamine therapy.

An important novelty of our study was the progressive increase of blood eosinophils in patients with CS. Eosinophils have recently emerged as key regulators of local immunity and remodeling/repair, critical for the resolution of inflammation and tissue repair^20^. Therefore, the delayed increase in blood eosinophils could represent an adaptation to the severe inflammation and tissue injury at the acute phase of CS. This would be in line with a recent study reporting that a reduction of blood eosinophils correlated with negative outcomes in patients with myocardial infarction^21^.

The main signals involved in the regulation of eosinophil mobilization and trafficking involve the chemokine eotaxin (CCL11) and the cytokine IL-5^22^. Eotaxin is a CC chemokine acting on the CCR3 receptor, which is critical for the recruitment of eosinophils in various inflammatory diseases^23^. We found that plasma eotaxin levels were highest at T1 and progressively decreased thereafter. This suggests that the generation of this eosinophil chemoattractant may represent an early counter-regulatory signal paving the way for a secondary, eosinophil-dependent reparative response. IL-5 is a member of the Th2 cytokines, together with IL-4, IL-9 and IL-13, which are signatures of type 2 immunity, an adaptive response involved in allergic inflammation, wound healing and control of metabolic homeostasis^20^. Furthermore, IL-5 is specifically responsible for eosinophil differentiation and survival^22,24^. The values of IL-5 remained below control values at all time-points, but displayed a progressive and significant increase over time. This pattern indicates that type 2 immune response is suppressed at the acute inflammatory phase, but may be progressively set in motion at the delayed phase of CS. This suggests that the initial predominant pro-inflammatory response may progressively shift towards a more anti-inflammatory and reparative type 2 response, which may be, at least in part, dependent on the recruitment of eosinophils. Additional studies exploring the time-course of type 2 cytokines in CS need to be performed to address this hypothesis.

An important feature of CS is a high rate of infectious complications^5,6^, which was also evidenced in the current study, with an infectious complication occurring in 62% of patients, which could represent an important confounding factor in the interpretation of the immune response associated with CS. However, we did not find major differences between patients with or without infection, the formers displaying only higher levels of IL-6 and lower monocyte counts at T2. Therefore, the presence of infection was only associated with a slight increase of the early pro-inflammatory response (higher IL-6 at T2), but did not change the global picture of the immune response.

Proposed hypotheses to explain the high rate of infections in CS include intestinal bacterial translocation, the use of indwelling catheters and endotracheal tube, as well as the need for mechanical ventilation^2,5,6^. Our findings suggest that impaired antimicrobial defenses could represent an additional mechanism, as it has been shown that the development of a type 2 immune response could promote some form of immune depression in unrelated pathological states including septic shock^25^, and stroke^26^. Furthermore, we found that lymphocytes were significantly reduced in patients with the highest SOFA scores (at T2, and to a lesser extent at T3) and norepinephrine doses (at T3), suggestive of impaired cell-mediated immune defenses in the most severely affected patient, which would be consistent with previous findings showing negative prognostic implications of lymphopenia in myocardial infarction^27^ and chronic heart failure^28^. We also noticed that higher norepinephrine doses and SOFA were associated with lower monocyte counts at T2 and T3, respectively, which could also point to some impairment of antimicrobial defenses, as we found that monocytes were lower at T2 in patients with infections. However, this observation should be interpreted with caution, given the opposite finding of a slightly higher monocyte count at T1 in the presence of infection.

Our study has several limitations. The first and main limitation is related to the small sample size, which implicates that our study may have been insufficiently powered to detect significant changes for several variables, and we also could not use multivariate analyses to detect some independent predictors for some important outcomes (such as mortality or the incidence of infectious complications). Second, in the absence of a control group of heart failure patients without shock, we cannot quantify the magnitude of the changes of inflammatory markers due exclusively to the circulatory failure in comparison to heart failure itself. Third, we investigated patients with CS due to different etiologies, and we cannot rule out that some observations may have been influenced by the initial mechanism responsible for CS, notably myocardial ischemia. Fourth, the mortality in our cohort was relatively low (21%) as compared to reported CS mortality (27–51%). It is therefore possible that patients with more severe forms of CS and higher mortality could behave differently on an immune standpoint. Therefore, our work should be considered as a pilot investigation, and future studies, involving more patients and complementary mechanistic investigations will be necessary to determine in more detail the immune consequences of cardiogenic shock.

In conclusion, our results indicate that CS is associated with an immediate, prototypical inflammatory response, which correlates with the severity of cardio-circulatory failure, and with a delayed increase in circulating eosinophils. Furthermore, in the most severe cases of CS, a rapid depletion of circulating immune cells was detected. Overall, these findings indicate that CS elicits complex immune alterations, ranging from an early pro-inflammatory phenotype, to a delayed phenotype more consistent with inflammatory resolution and immune depression in the most severely affected patients.

## Methods

### Patients

From June 2017 to November 2018, we prospectively included 24 patients hospitalized in our 35-bed multidisciplinary intensive care unit (ICU), with a diagnosis of cardiogenic shock of various etiologies. The study conforms with the principles outlined in the *Declaration of Helsinki*, and was approved by our local ethical committee, the Commission cantonale d'éthique de la recherche sur l'être humain (*CER*-*VD*), authorization Nr 2017-00345. Informed consent was obtained from the patient or his/her next of kin. Inclusion criteria were cardiogenic shock, defined according to standard criteria of arterial hypotension requiring catecholamines in the absence of hypovolemia, with one or more signs of systemic hypoperfusion (cold/clammy skin and extremities, low urine output, altered mental status, lactic acidosis)^29^, associated with echocardiographic signs of left ventricular dysfunction (LVEF ≤ 35%) and/or right ventricular dysfunction. Exclusion criteria included moribund patient, immunosuppression (HIV infection and immunosuppressive therapy), Child C cirrhosis, CS post-heart transplantation, pregnancy and breast feeding.

### Measurements

The severity scores SAPS 2 and SOFA (Sequential Organ Failure Assessment) for the first 24 h were calculated. Systemic blood pressure was monitored with an intra-arterial catheter (femoral or radial artery). The implementation of invasive hemodynamic monitoring by a pulmonary artery catheter (PAC) or transpulmonary thermodilution (PiCCO® technology) was at the discretion of the physicians in charge. All episodes of secondary infections were determined. The primary site of infection, the results of microbiological cultures, the duration of antibiotic therapy and the delay between the admission and the initiation of antibiotherapy were recorded.

Three sequential blood samples, at day 1 (within 12 h of hospital admission and labeled T1), day 3 (48 h after T1, labeled T2), and day 6-8 (labeled T3) were obtained to determine complete blood count with differential counts of WBCs, plasma levels of liver aminotransferases, creatinine, CRP, procalcitonin, troponin T, NT-pro-BNP, glucose, arterial pH and lactate, and the cytokines Interleukin (IL)-1 beta (IL-1β), IL-5, IL-6, IL-10, Interferon gamma (IFNγ), tumor necrosis alpha (TNFα), monocyte-chemoattractant protein-1 (MCP-1) and eotaxin (CCL-11), an eosinophil-specific chemoattractant. Plasma cytokines were measured at the Mouse Metabolic Evaluation Facility, University of Lausanne, using a Merck Millipore Milliplex^®^ Human Cytokine Kit (HCYTOMAG-60K-08) in a Luminex^®^200 system and following the manufacturer’s procedure, except from eotaxin, which was measured with a commercially available kit (Eotaxin Human ProQuantum Immunoassay Kit®, ThermoFisher Scientific, Reinach, Switzerland). Plasma samples from 30 hemodynamically stable cardiac patients scheduled for cardiac surgery and taking part to an unrelated study^30^ were used as controls for the measurement of cytokines, with the only differences that samples were collected in EDTA-coated tubes and not in lithium-heparin-coated tubes as in the current study. Plasma eotaxin was the only cytokine which was not measured in this control group.

### Statistical analysis

Continuous variables are expressed as medians and interquartile range. Categorical variables are shown as absolute numbers and percentages. The time-course (T1, T2, T3) of hemodynamic data, plasma chemistry variables and WBCs were evaluated by one way ANOVA, followed by Tukey test to compare values at T2 and T3 with T1. For plasma cytokines, we performed comparisons between the initial value (T1) with both T2 and T3 values using non-parametric Wilcoxon rank-sum test, while comparisons with the control group were made using Dunnett’s test. In a subgroup analysis, we compared values of WBCs, CRP and cytokines at each time-point between patients with or without infection and antibiotic treatment, using one way ANOVA. To explore associations between cytokines, mean BP and catecholamine treatment, we performed linear regression analyses with calculation of the Pearson’s r coefficient and r square coefficient of determination. To evaluate possible influences of the severity of CS on circulating immune cells (lymphocytes and monocytes), we considered 2 indicators of severity, including the SOFA score and the intensity of norepinephrine treatment in the first 24 h. Two groups of patients (above or under the median value of each indicator) were compared with respect to the numbers of lymphocytes and monocytes at T1, T2 and T3, using non-parametric Wilcoxon rank-sum test. For all statistics, a p value of less than 0.05 was considered significant. We used the JMP statistical software, version 13 for the analyses.

## Supplementary information

Supplemental Table S1.

## Data Availability

All data generated or analyzed during this study are included in this published article (and its Supplementary Information File).
