# Supplementary material for: Cardiogenic shock elicits acute inflammation, delayed eosinophilia, and depletion of immune cells in most severe cases
Source: Sci Rep. 2020 May 6;10:7639. doi: 10.1038/s41598-020-64702-0 (PMC7203157; doi:10.1038/s41598-020-64702-0)
Supplement: Supplementary file 1 — Supplemental Table S1. [file 41598_2020_64702_MOESM1_ESM.docx]

**Cardiogenic shock elicits acute inflammation, delayed eosinophilia, and depletion of immune cells in most severe cases.**

Justine Cuinet, MD^1^, Andrea Garbagnati, MD^1^, Marco Rusca, MD^1^, Patrick Yerly, MD^2^, Antoine G. Schneider, MD^1^, Matthias Kirsch, MD^3^, and Lucas Liaudet, MD^1^*

^1^Service of Adult Intensive Care Medicine, ^2^Service of Cardiology, ^3^Service of Cardiac Surgery, University Hospital Medical Center and Faculty of Biology and Medicine, Lausanne, Switzerland

*** Corresponding author**

Lucas Liaudet, MD

ORCID: 0000-0003-2670-4930

Service of Adult Intensive Care Medicine

University Hospital, Rue du Bugnon 46

Lausanne 1011, Switzerland

Phone: +4121 3140514. Fax: +4121 3143045. E-mail: lucas.liaudet@chuv.ch

Supplemental Table S1. WBCs and cytokines, according to infection and antibiotic treatment

Variable Time No Antibiotics Antibiotics

WBCs (G/L) T1 15.5 (11.3) 15.8 (5.6)

T2 10.2 (5.0) 11.3 (5.4)

PMNs (G/L) T1 15.4 (9.9) 11.1 (8.0)

T2 8.4 (4.8) 8.5 (4.5)

Monocytes (G/L) T1 0.9 (1.9) 1.0 (0.7)^a^

T2 1.5 (1.3) 0.7 (0.6)*

Lymphocytes (G/L) T1 1.2 (1.9) 1.3 (1.2)

T2 1.1 (1.0) 1.0 (1.2)

Eosinophils (G/L) T1 0 (0.18) 0 (0)

T2 0 (0.14) 0 (0.19)

IL-1 β (pg/mL) T1 0.87 (0.86) 0 (0.56)

T2 0.66 (2.01) 0.09 (0.84)

IL-6 (pg/mL) T1 58 (92) 36 (63)

T2 27 (31) 70 (94)^a^

IFN γ (pg/mL) T1 4.0 (12.0) 2.6 (2.1)

T2 1.1 (10.7) 1.8 (9.3)

MCP-1 (pg/mL) T1 655 (455) 1361 (1868)

T2 534 (429) 1130 (1263)

TNF α (pg/mL) T1 46 (22) 42 (24)

T2 35 (11) 43 (40)

IL-10 (pg/mL) T1 24 (373) 28 (147)

T2 11 (10) 16 (33)

IL-5 (pg/mL) T1 0.6 (0.9) 0.5 (0.7)

T2 0.6 (0.7) 0.9 (1.1)

For all measurements, antibiotics: n=17 (T1, T2); no antibiotics: n=7 (T1), n=6 (T2)

Data at T3 not shown (only 1 patient at T3 in the no antibiotics group).

Data are given as medians (IQR). ^a^ p=0.05; * p<0.05 (one way ANOVA)
